# Supplementary material for: Toward three-dimensional hybrid inorganic/organic optoelectronics based on GaN/oCVD-PEDOT structures
Source: Nat Commun. 2020 Oct 9;11:5092. doi: 10.1038/s41467-020-18914-7 (PMC7547673; doi:10.1038/s41467-020-18914-7)
Supplement: Supplementary file 1 — Supplementary Information [file 41467_2020_18914_MOESM1_ESM.pdf]

Supplementary Information

Toward three-dimensional hybrid inorganic/organic optoelectronics based on GaN/oCVD-PEDOT structures

Linus Krieg[1,7], Florian Meierhofer[1,7], Sascha Gorny[1], Stefan Leis[1], Daniel Splith[2], Zhipeng Zhang[2], Holger von Wenckstern[2], Marius Grundmann[2], Xiaoxue Wang[3,4], Jana Hartmann[1,5], Christoph Margenfeld[1,5], Irene Manglano Clavero[1,5], Adrian Avramescu[6], Tilman Schimpke[6], Dominik Scholz[6], Hans-Jürgen Lugauer[6], Martin Strassburg[6], Jörgen Jungclaus[1], Steffen Bornemann[1,5], Hendrik Spende[1,5], Andreas Waag[1,5], Karen K. Gleason[3], Tobias Voss[1**]

[1] Institute of Semiconductor Technology and Laboratory for Emerging Nanometrology, Tech-nische Universität Braunschweig, Langer Kamp 6a/b, 38106 Braunschweig, Germany.

[2] Felix-Bloch-Institut für Festkörperphysik, Universität Leipzig, Linnéstraße 5, 04103 Leipzig, Germany.

[3] Department of Chemical Engineering, Massachusetts Institute of Technology, 77 Massachu-setts Avenue, Cambridge, MA 02139, United States.

[4] Department of Chemical and Biomolecular Engineering, Ohio State University, 151 W. Wood-ruff Avenue, Columbus, OH 43210, United States

[5] Epitaxy Competence Center ec[2], Technische Universität Braunschweig, Hans-Sommer- Str. 66, 38106 Braunschweig, Germany.

[6] OSRAM Opto Semiconductors GmbH, Leibnizstr. 4, 93055 Regensburg, Germany

[7] These authors contributed equally: Linus Krieg, Florian Meierhofer.

* tobias.voss@tu-braunschweig.de

Supplementary Information

Supplementary Figures

Supplementary Note 1: Electrical characterization of planar reference structures

Supplementary Note 2: Additional information about contacts and breakdown voltages

Supplementary Note 3: Determination of temperature-independent constants of proportionality within the model of Werner and Güttler

Supplementary Note 4: Determination of homogeneous barrier heights according to the empirical model of Schmitsdorf et al.

Supplementary Methods: Sample Fabrication

Supplementary References

Supplementary Figures

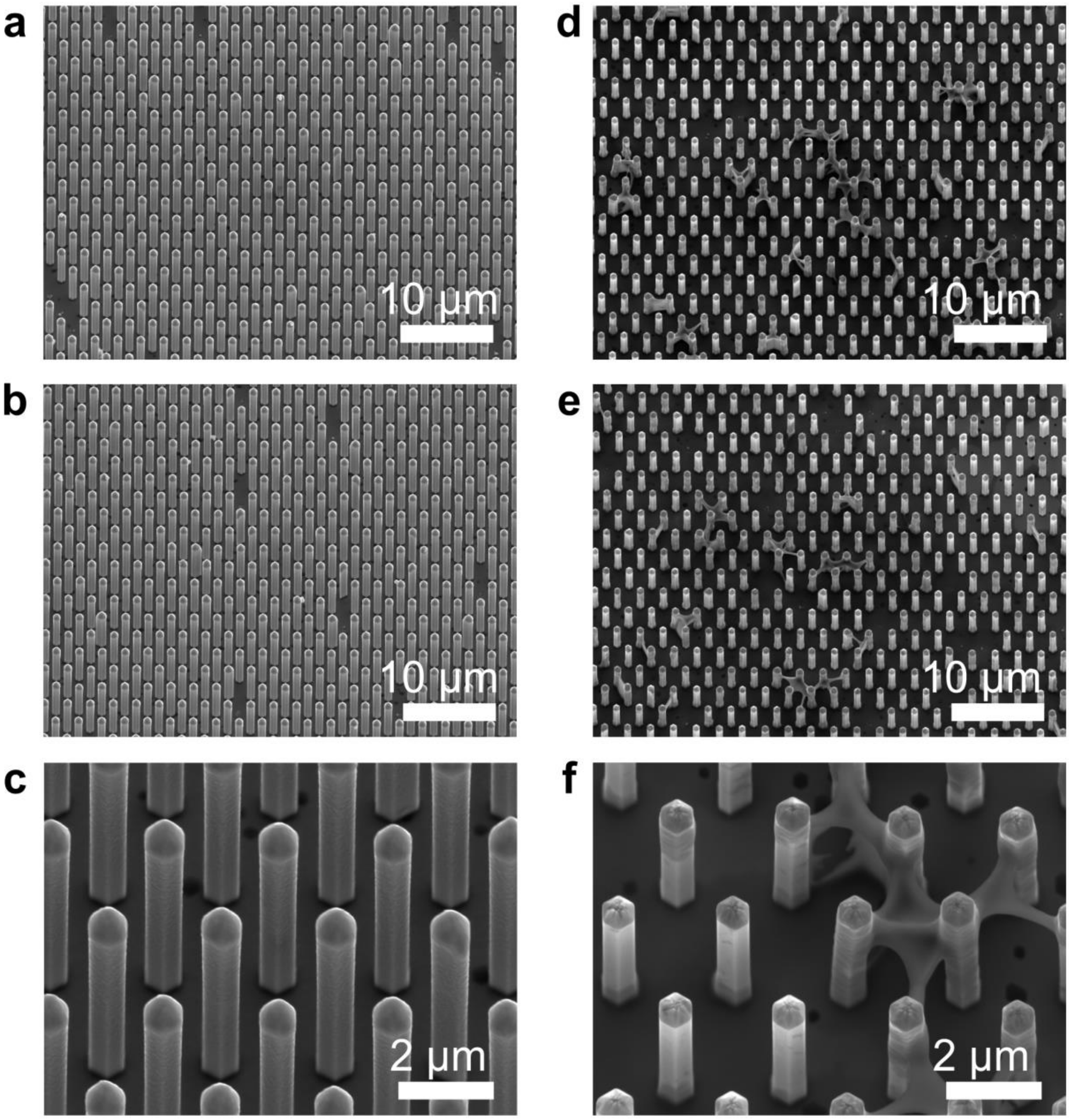

Supplementary Fig. 1: SEM micrographs of additional microrods coated with PEDOT. a-c Microrods coated with PEDOT using oCVD. The polymer layer around the rods can be seen around the whole structure (secondary electron map, tilt = 30°, E_{PE} = 5 kV). **d-f** Microrods coated with PEDOT:PSS using spin-coating (secondary electron map, tilt = 15°, E_{PE} = 5 kV). Scale bars in **a**, **b**, **d** and **e** are 10 µm, scale bars in **c** and **f** are 2 µm.

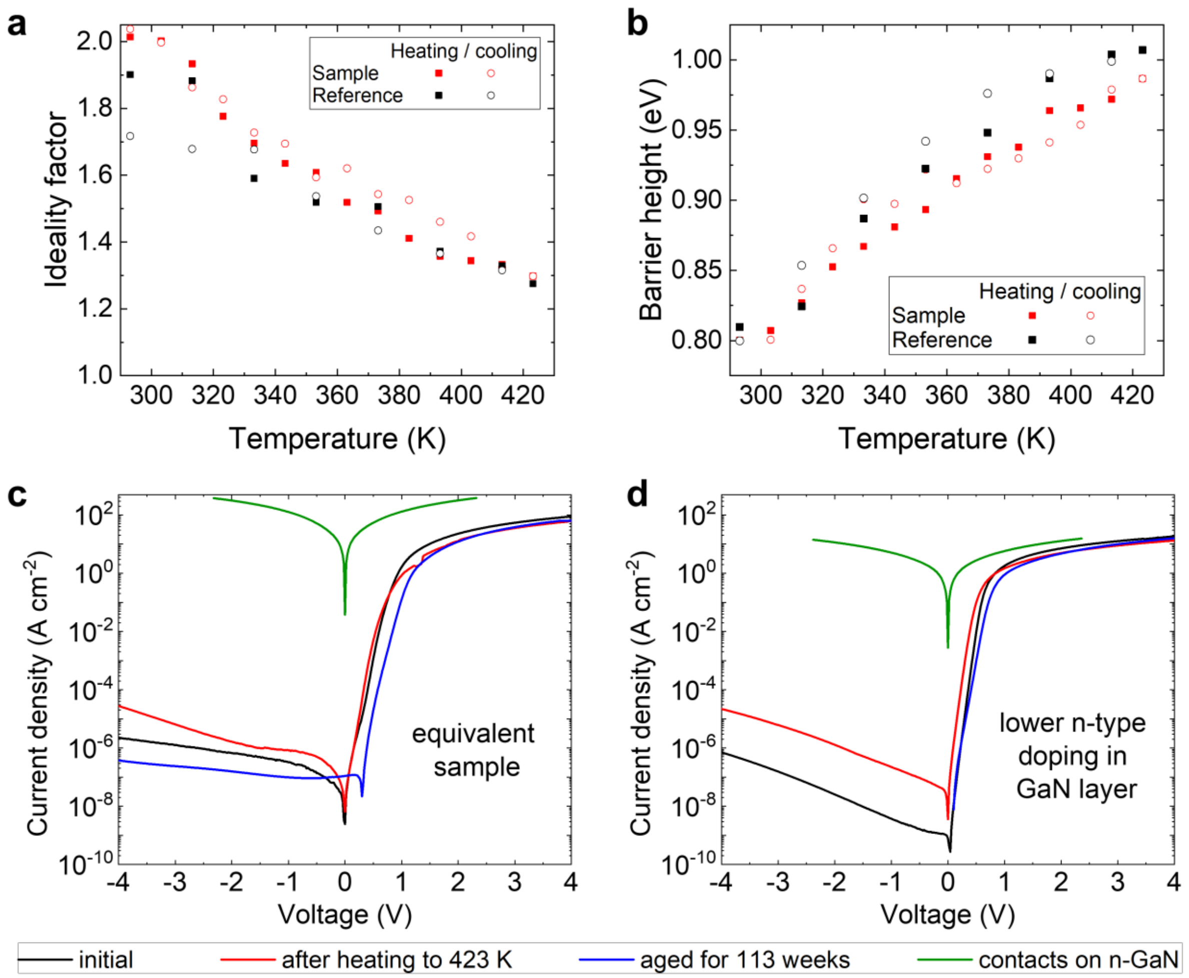

Supplementary Fig. 2: Batch reproducibility of hybrid planar PEDOT/n-GaN samples. a, b Ideality factors and barrier heights obtained for the sample under investigation and its equivalent reference sample from temperature-dependent I-V measurements. **c, d** Electrical characteristics obtained for the equivalent reference sample to the sample discussed in the article and a sample, where the n-type doping of the GaN layer is one order of magnitude lower (2 x 10^{17} cm^{-3} instead of 2 x 10^{18} cm^{-3}).

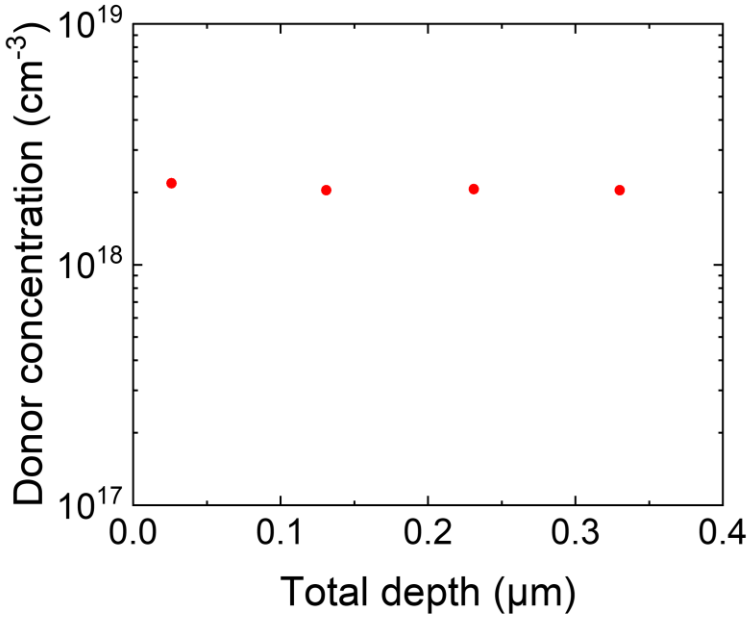

Supplementary Fig. 3: Donor concentration of GaN. A constant donor concentration in the n-GaN layer used for the hybrid PEDOT/n-GaN junction presented in the main article is confirmed by electrochemical capacitance-voltage (ECV) analysis.

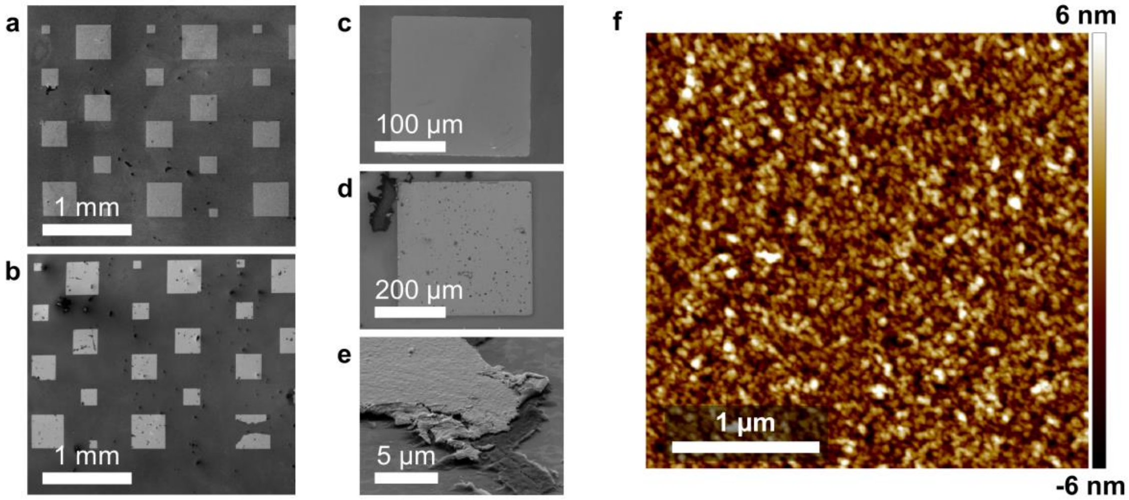

Supplementary Fig. 4: SEM micrographs of various contacts on the planar PEDOT/n-GaN sample and AFM micrograph of a PEDOT layer. a SEM image of an Au/Ti/n-GaN and **b** of an Au/PEDOT/n-GaN array (scale bars: 1 mm). Whilst the contact pads without polymer are highly regular, the pads with a PEDOT interlayer show damages that are assigned to the growth process. **c** Close-up of an Au/Ti/n-GaN contact (scale bar: 100 µm) and **d** of an Au/PE-DOT/n-GaN contact (scale bar: 200 µm). **e** SEM micrograph of an Au/PEDOT structure (scale bar: 5 µm): The scratched contact pad shows that a closed PEDOT layer forms between the Au top contact and the GaN thin film (secondary electron map, tilt = 0° for **a**-**d**, tilt = 60° for **e**, E_{PE} = 5 kV). **f** AFM micrograph of an oCVD PEDOT layer grown at comparable growth conditions (T_{Subs} = 150°C). The surface roughness of this layer is measured to be 2 nm (scale bar: 1 µm).

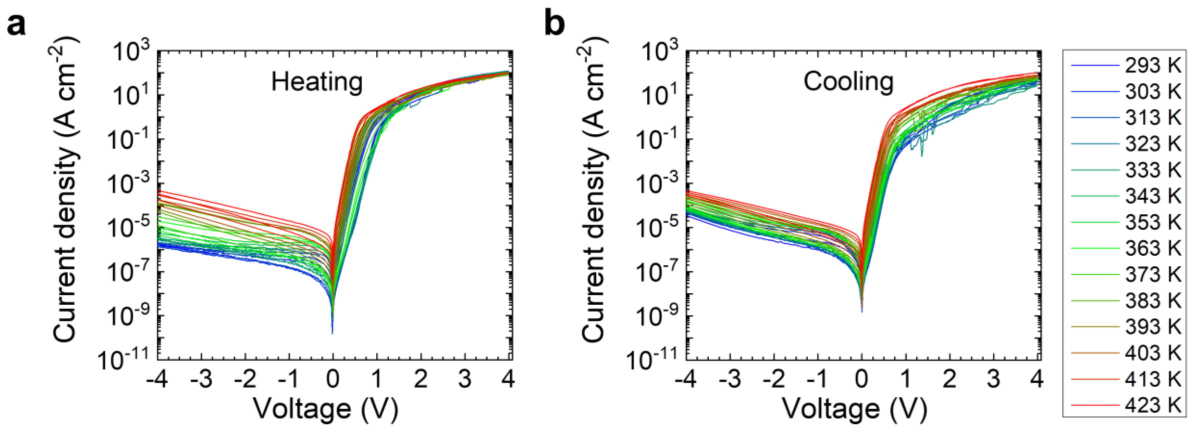

Supplementary Fig. 5: Thermal stability of the electrical characteristics. a I-V characteristics of the PEDOT/n-GaN junction during heating and **b** cooling in the temperature range of 293-423 K, respectively. The overall characteristics are maintained. In reverse direction, the characteristics do not reach the initial values.

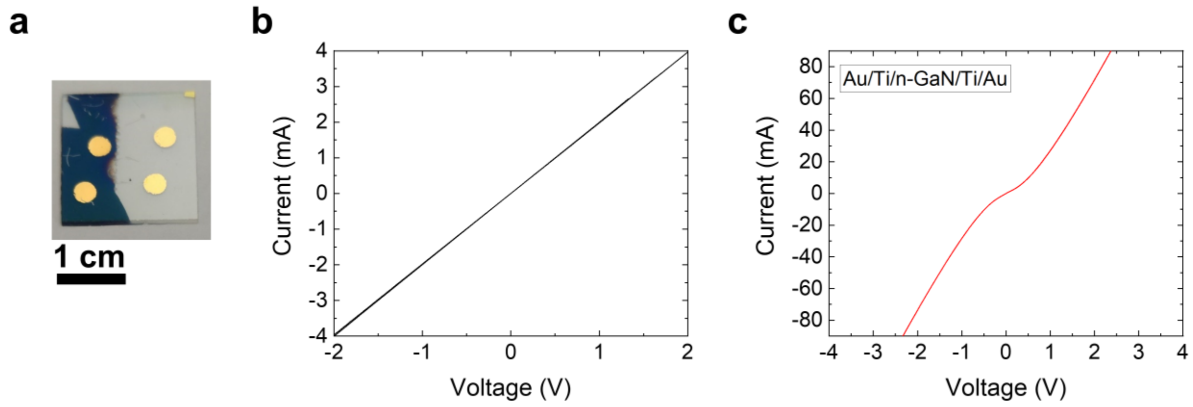

Supplementary Fig. 6: Electrical characterization of the contact properties. a Photograph of a different planar Au/n-GaN/PEDOT/Au sample. The blueish area on the left is the n-GaN/PEDOT region, on the right-hand side, the uncovered n-GaN area is visible (scale bar: 1 cm). **b** The I-V characteristic of the Au contacts on PEDOT in the structure shown in A yield a linear behavior indicating ohmic contacts for Au deposited on PEDOT. **c** I-V measurements of the Au/n-GaN contacts of the sample discussed in this article yield deviations from the expected linear behavior of the contacts. However, this does not influence the overall performance of the hybrid device as the rectification from the contacts plays a role for higher voltages only (compare Fig. 2c).

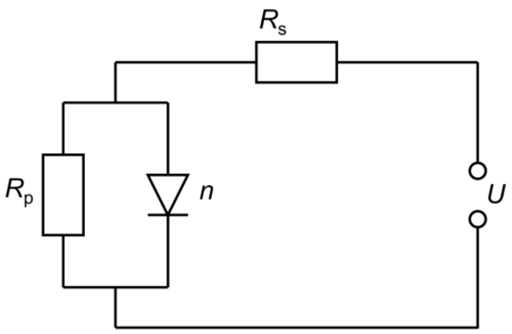

Supplementary Fig. 7: Equivalent circuit underlying the model for fitting the I-V characteristics.

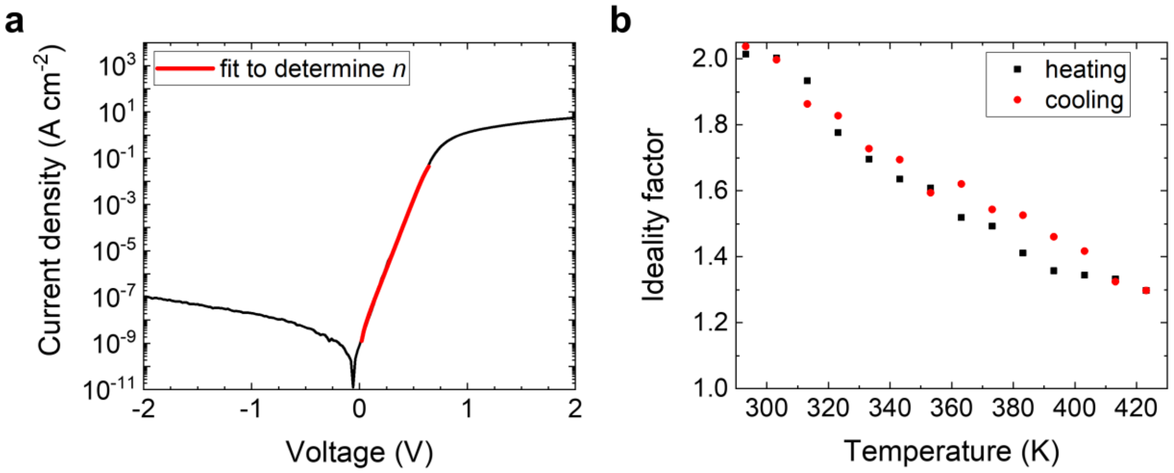

Supplementary Fig. 8: Determination and temperature dependence of ideality factors. **a** Ideality factors were obtained by using a linear fit to the slope in forward direction of the semi-logarithmic I-V curves. **b** Ideality factors obtained for different temperatures during the heating (black) and cooling (red) process.

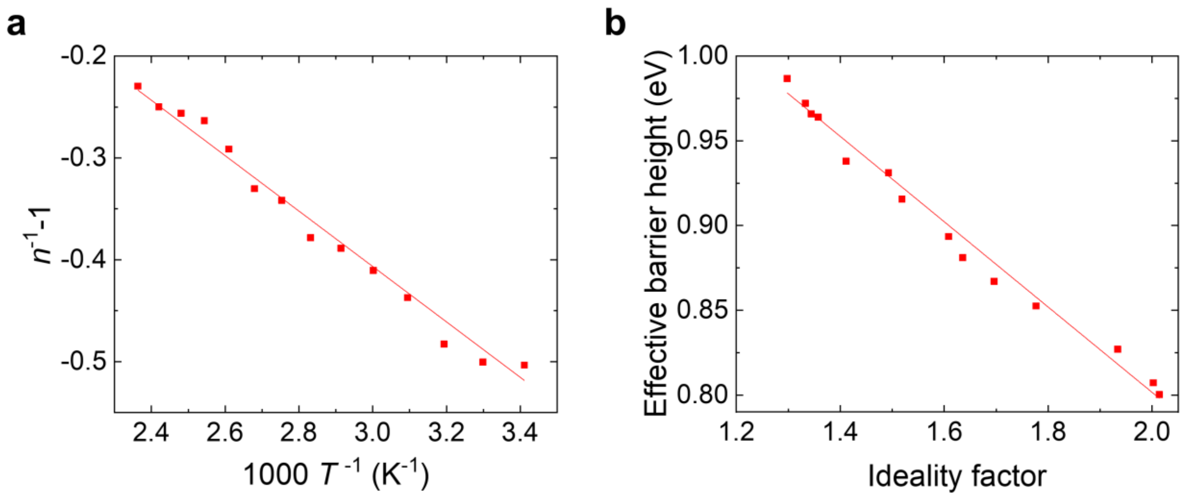

Supplementary Fig. 9: Further evaluation of model parameters. a The linear dependence of the inverse ideality factors on the inverse temperature is in agreement with the potential fluctuation model of Werner and Güttler according to Supplementary Equation 1 as indicated by the linear fit. **b** Effective barrier height as a function of the ideality factor determined for one diode at different temperatures. According to the model of Schmitsdorf et al[1], interpolation of the linear dependence to n = 1.02 yields a homogeneous barrier height of (1.05 ± 0.02) eV.

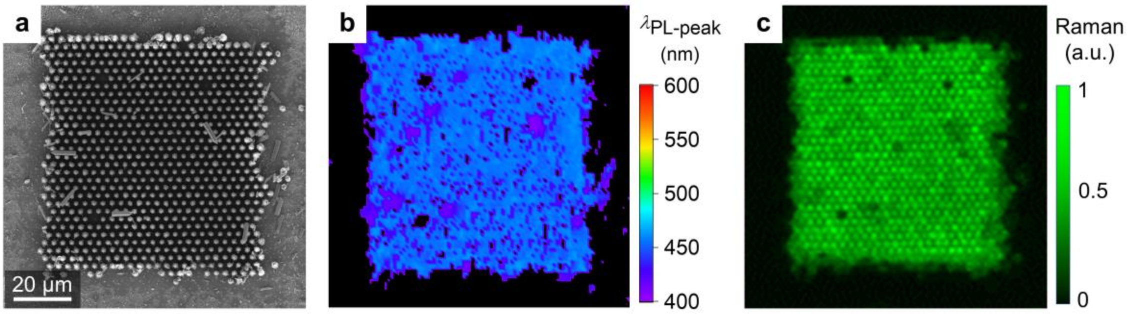

Supplementary Fig. 10. **Patterning of the hybrid 3D microrod LED array. a** SEM image of a microrod array coated with oCVD PEDOT. Laser ablation removed all microrods and the oCVD PEDOT outside the array as can be seen by **b** PL and **c** Raman mapping (scale bar: 20 µm). The InGaN MQW luminescence around 450 nm is only detected in the region of the sample where microrods are visible in the SEM image. We note that the PL map shows the wavelength corresponding to the most intense PL line. In connection with the SEM, we can therefore clearly distinguish between the array of undamaged microrods and voids as well as broken microrods, which show a luminescence corresponding to GaN below 400 nm. Similarly to the PL mapping, Raman spectroscopy identifies the PEDOT coating just on the microrod array. The color code of the Raman map is based on the normalized integrated intensity of the characteristic signals of PEDOT obtained in the range of 800 to 1400 cm^{-1}.

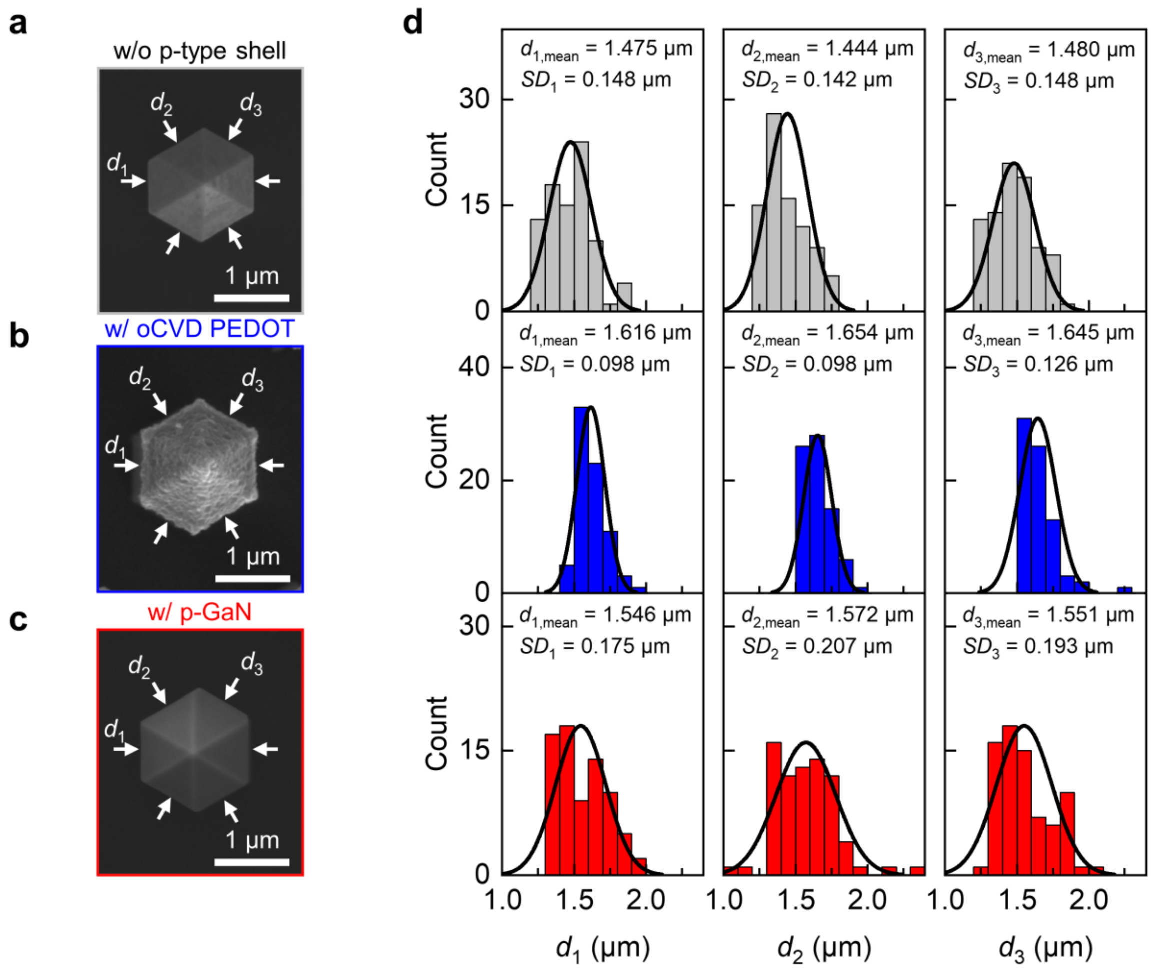

Supplementary Fig. 11: Thickness of the p-type shell. a SEM top view of a microrod before, **b** after oCVD PEDOT coating and **c** with a p-GaN shell (scale bars: 1 µm). Based on the six m-plane surfaces of the hexagonal rod structure, the distances d_1, d_2 and d_3 are measured and **d** statistically evaluated based on arithmetic mean values and standard deviations (SD). An average PEDOT thickness of 86 ± 14 nm is obtained by comparing rods with and without oCVD coating. Similarly, the thickness of the p-GaN is estimated to be 45 ± 14 nm.

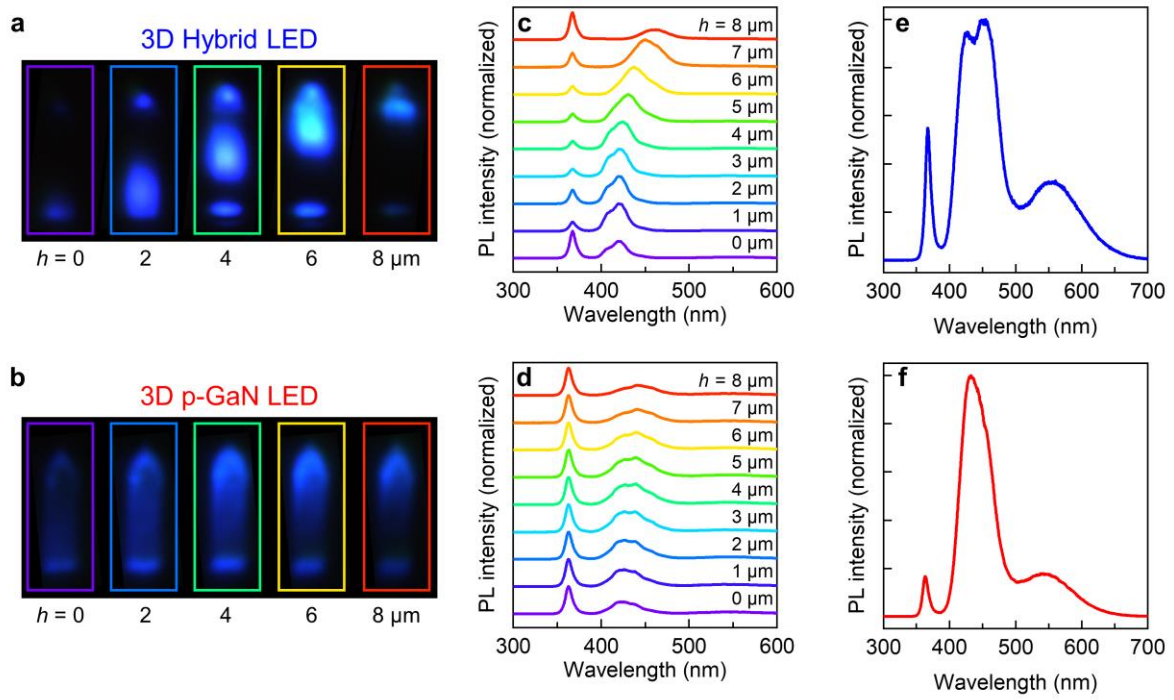

Supplementary Fig. 12: Photoluminescence of 3D core-shell microrod hybrid and p-GaN-based LED structures. a PL spectra recorded along the rod height *h* of a single hybrid and **b** reference microrod LED. **c, d** Both LED designs show a red shift of the InGaN luminescence along the rod height which is attributed to an inhomogeneous concentration of indium inside the MQW (more In at the top). The maxima of the PL spectra of the **c** hybrid and the **d** purely inorganic rods shift from ~420 to ~460 nm and from ~421 to ~443 nm, respectively. This is in reasonable agreement with the shifts obtained from CL analysis (*cf*. Figs. 5c-f). For the purely inorganic microrod the p-GaN shell contributes a continuous PL-signal over the entire height of the microrod which exceeds the InGaN luminescence in the PL spectra. **e**, **f** PL spectra of microrod arrays excited from the top for the hybrid and the purely inorganic microrod structure. The signals around 365 nm and 560 nm correspond to the near band edge emission and the well-known yellow luminescence, respectively. The InGaN emission is centered around 440 nm with two distinct maxima at 425 and 450 nm in the case of the hybrid structure.

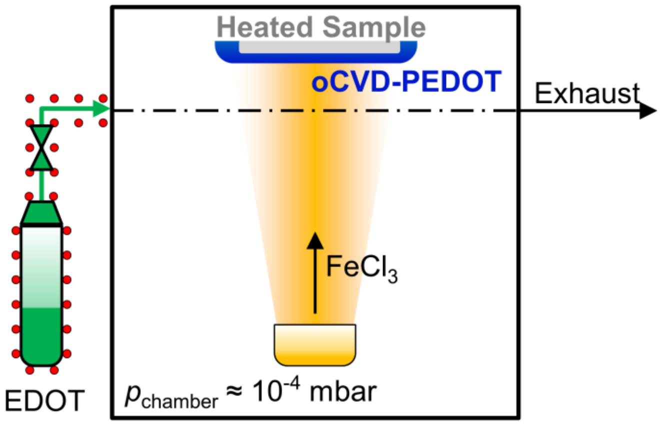

Supplementary Fig. 13: Sketch of the oCVD reactor. The sample is mounted face down on a heated stage in the top region of the vacuum chamber. The monomer (EDOT) flows into the reactor while the oxidizing agent ($FeCl_3$) is co-sublimated. The polymerization persists as long as the monomer and the oxidizing agent are provided.

Supplementary Note 1: Electrical characterization of planar reference structures

To ensure batch reproducibility, several planar GaN samples were placed inside the oCVD vacuum chamber for the oCVD PEDOT deposition. Therefore, we were able to characterize the sample presented in the article as well as two additional samples. One is a replica (Supplementary Fig. 2a-c), also described as reference sample, which was processed and characterized in the same way as the sample presented in the main article. The other sample (Supplementary Fig. 2d) has a lower doping concentration of the GaN layer (2 x 10^{17} cm^{-3}) as compared to the sample presented (2 x 10^{18} cm^{-3}). This allows us to determine the influence of the GaN layer on the electrical characteristics. The electrical measurements on the reference sample (Supplementary Fig. 2c) reproduce well the characteristics of the original sample (Fig. 2c). The thermal stability after heating to 423 K and the temporal stability after aging for 113 weeks at ambient conditions in darkness as compared to the initial measurement is significant. Differences can be seen in backwards direction where the heating process enlarged the current density. In forward direction, we see a slightly larger current density. This can be attributed to a different diode (PEDOT patch) measured on the sample as compared to the initial diode. The contacts on GaN showed a high current density which is larger than the current density of the device, indicating that the impact of the contacts on the hybrid junction can be neglected. Supplementary Figs. 2a and 2b compare the ideality factors and barrier heights as obtained for temperature series for the original sample and its equivalent. Once again, the obtained data is comparable. Whilst for the original sample a mean barrier height of (1.42 ± 0.01) eV was determined using the model of Werner and Güttler[2], the equivalent sample reaches a value of (1.49 ± 0.03) eV. Hence, even though these values deviate slightly, they are in the same order and consistent.

The electrical characteristics of the sample with a lower doping density are presented in Supplementary Fig. 2d. Once again the good thermal and temporal stabilities are demonstrated. Additionally, we note, that the maximum current density of the contacts of both samples in forward direction in Supplementary Figs. 2c and 2d differ by about an order of magnitude (318 A cm^{-2} vs. 13 A cm^{-2} at +2 V), which reflects the difference in doping concentrations of the

n-GaN thin film (2 × 10^{18} cm^{-3} vs. 2 × 10^{17} cm^{-3}). This supports the observation, that the current density is limited by the conductivity of the n-GaN rather than the conductivity of PEDOT within the devices.

Supplementary Note 2: Additional information about the contacts and breakdown voltages

To rule out contributions from the contacts on the rectification of the hybrid planar structure, I-V measurements of Au/Ti/n-GaN/Ti/Au (2 contacts without PEDOT interlayer) were performed. As depicted in Supplementary Fig. 6c, an s-shape behavior in the I-V characteristic indicates deviations from an ohmic behavior of the contacts. However, as the overall current density for the contacts is much larger than the current densities for the hybrid devices (Fig. 2c), influences of the contacts on the rectification of the full hybrid structure in the voltage range under investigation can be neglected. I-V measurements of Au/PEDOT/Au-junctions on earlier samples (Supplementary Fig. 6a) showed ohmic characteristics (Supplementary Fig. 6b). However, due to the patterned sample design, no such measurements could be performed on the sample analyzed and depicted in this article.

The breakdown voltage for the sample presented was determined in I-V measurements which were performed starting from 0 V and going down to end values from -4 V to -12 V with stepwise increments of -1 V. Between every measurement, the hybrid junction was measured from -4 V to 4 V to ensure the overall electrical characteristics remained unchanged. Fig. 2d shows the characteristics for negative bias voltages. The breakdown voltage for this sample is determined to about -7.8 V. This breakdown was reversible and the characteristic returned to the previously recorded values. At a voltage of around -10.8 V, a hard irreversible breakdown is reached. The rectification of the device is lost for all following measurements indicating the formation of ohmic channels or shunts through the device. A measurement at a comparable structure yielded -8.2 V and -11.4 V for soft and hard breakdown, respectively, thus confirming that these values are characteristic for the studied hybrid structures.

Contact processing is a crucial factor for device fabrication. The n-contacts of our structures are processed by using RIE-ICP and subsequent KOH healing prior metallization of Au/Ti which is well established for n-GaN contacts. For the p-contacts, we can picture several optimization steps. These steps comprise annealing of the Au contacts in an inert atmosphere or low-temperature deposition of the Au contact. The structuring of PEDOT can be challenging and might negatively impact the process yield (number of suitable pn-junctions on the wafer). In the present study, we attempted the lift-off approach and obtained several desired square-shaped p-contacts of PEDOT/Au (cf. Fig 2b) with a high IV reproducibility. For the hybrid LED devices we moved on to structure the polymer layer using laser ablation. The advantages of laser ablation can be seen in Supplementary Fig. 10. With the help of SEM as well as PL and Raman spectroscopy, laser ablation removes GaN and PEDOT reliable without contamination of any surface through photoresists or shadow masks. The strength is also demonstrated in Fig. 4b and 4f for planar polymer layers that follow a well-defined pattern due to laser ablation.

Supplementary Note 3: Determination of temperature-independent constants of proportionality within the model of Werner and Güttler

In the case of voltage-independent ideality factors, their temperature dependence can be explained if the mean barrier $\Phi_{B,m}$ and the square of the standard deviation σ^2 depend linearly on the applied bias U[2]:

$$\phi_{\mathrm{B,m}} = \phi_{\mathrm{B0},m} + e\rho_2 U \qquad (1)$$

$$\sigma^2 = \sigma_0^2 + e\rho_3 U \qquad (2)$$

σ_0 is the standard deviation at zero bias, ρ_2 and ρ_3 are constants of proportionality that quantify the deformation of the local barrier heights under an applied voltage. In the case of temperature-independent constants of proportionality ρ_2 and ρ_3, the ideality factor can be described as

$$n^{-1}(T) - 1 = -\rho_2 + \frac{\rho_3}{2k_{\mathrm{B}}T} \ . \qquad (3)$$

Therefore, plotting $1/n - 1$ versus $1/T$ should yield a straight line. Such a linear dependency can clearly be seen for our sample (Supplementary Fig. 9a) thus confirming the consistency

with the model. A linear fit yields proportionality constants of ρ_2 = -0.41 ± 0.03 and ρ_3 = (-47 ± 2) meV.

Supplementary Note 4: Determination of homogeneous barrier heights according to the empirical model of Schmitsdorf et al.

Schmitsdorf et al.[1] presented an approach to determine homogeneous barrier heights $\Phi_{B0,h}$ of Schottky barriers based on a theoretical model of Tung et al.[3] They considered the Schottky contacts as nonuniform or "patchy" in analogy to models with separate diodes with different barrier heights and areas in parallel. The difference to a model with separate diodes lies in the size of the patches that can be in the order of or even smaller than the Debye length. Also, the patches of density ρ are embedded in the contact. The barrier height of the patches is lower than the barrier height of the otherwise uniform interface. Therefore, the homogeneous barrier height of the surrounding interface is reduced. The barrier height of the homogeneous interface can be determined by relating the effective barrier height to the ideality factor, which, in the case of the sample discussed in this article returns a linear dependence (Supplementary Fig. 9b). Interpolating the linear fit to n = 1.02 which corresponds to the ideality factor describing Schottky barriers with image-force lowering only as reason for the bias dependence of the barrier height yields a homogeneous barrier height of $\Phi_{B0,h}$ = (1.05 ± 0.02) eV. This value is lower than the previously determined mean barrier height of $\Phi_{B0,m}$ (0 K) = (1.42 ± 0.01) eV using equation 3. Still, both the analytical approach by Werner and Güttler as well as the empirical model by Schmitsdorf et al. yield comparable barrier heights, even though the ideality factors obtained show values >1.5 for lower temperatures, where the empirical model starts to deviate from a linear dependence between ideality factor and effective barrier height.

Supplementary Methods: Sample Fabrication

Growth of GaN Specimens by Metal-Organic Vapor Phase Epitaxy (MOVPE): The investigated gallium nitride epilayers are grown by metal-organic vapor phase epitaxy on 430 µm thick 2" c-plane sapphire substrates using standard precursors trimethylgallium (TMGa), triethylgallium (TEGa), trimethylindium (TMIn), trimethylaluminium (TMAl), monosilane (SiH_4), magnesocene (Cp_2Mg) and ammonia (NH_3), as well as hydrogen and nitrogen as carrier gas. The LED specimen and GaN templates are manufactured in an Aixtron AIX2600HT G3 24×2" planetary reactor, while the growth of planar silicon-doped GaN buffers and GaN microrods is carried out in a Thomas Swan 3×2" close-coupled showerhead reactor.

For the growth of all planar samples, the sapphire wafers are thermally cleaned in hydrogen at up to 1100 °C prior to a nitridation step and the deposition of a low-temperature GaN nucleation layer. Following a recrystallization and coalescence step, the deposition of silicon-doped GaN occurs at a temperature of 1050 °C, reactor pressures between 125 and 290 mbar and a V/III (NH_3/TMGa) ratio of around 1000 using hydrogen as carrier gas.

n-GaN/p-PEDOT diodes are manufactured on a 4 µm thick GaN layer that is homogeneously doped with 0.6 and 6.0 nmol min^{-1} of silane, resulting in donor concentrations of 2×10^{17} and 2×10^{18} cm^{-3} as obtained by electrochemical capacitance-voltage profiling (ECV).

The planar LED sample consists of a 4.4 µm thick n-GaN stack with silicon concentrations varying between 1×10^{18} and 3×10^{19} cm^{-3} and an active region of 250 nm thickness, containing a fourfold InGaN multi-quantum well (MQW) emitting at 2.78 eV as obtained by photoluminescence measurements. The growth of the active region is carried out at low temperatures between 735 and 795 °C using TEGa as gallium source and nitrogen as carrier gas. After the growth of the active region, the carrier gas is switched back to hydrogen for the deposition of a 30 nm thick magnesium-doped $Al_{0.15}Ga_{0.85}N$ electron blocking layer and two 60 nm thick p-GaN layers at 950 °C. The magnesium concentrations in the p-side of the LED structure amount to $2x10^{20}$, $5x10^{19}$ and $2x10^{20}$ cm^{-3}, respectively. Concluding the growth, the magnesium acceptors are thermally activated in nitrogen ambient within the MOCVD reactor. For the hybrid

LED structure, the deposition is stopped after the deposition of the p-type electron blocking layer.

For the growth of GaN microrods by selective area MOVPE, GaN templates were masked with SiO_x which is structured with a hexagonal pattern of 0.8 µm circular openings and a pitch of 2.4 µm. During the epitaxy process, a low V/III ratio of 77 and a high SiH_4 flux of 37 nmol min^{-1} was applied for 20 min. The grown rods have six m-plane {1-100} sidewall facets and a tip consisting of semi-polar {1-102} facets. For all GaN microrods used in this work the height of the microrods is in the range of 3 to 8 microns with a diameter of about 1 micron.

The core-shell LED structures were fabricated by overgrowing GaN cores with a sequence of functional layers as follows. First, a thin quaternary n-AlInGaN layer was deposited on the highly n-doped cores to promote coverage of the Si-rich surface layer[4]. Afterward, a high-quality n-doped GaN shell layer was grown at temperatures higher than 1000°C and V/III ratios two orders of magnitude larger than those used during the growth of the vertical GaN rods. The active region, which consists of multiple InGaN quantum wells (QW), separated by GaN barrier layers were grown at moderate temperatures between 730°C and 830°C. Directly on the active region an AlGaN:Mg electron blocking layer (EBL) for improved carrier confinement was deposited. The growth temperatures for the p-type shell was set above 900°C to assure adequate electrical properties. The reference microrod structure has an additional Mg-doped p-GaN layer grown on top of the EBL. Due to the 3D nature of the structures, the exact amount of Mg incorporation in the p-AlGaN and p-GaN layers could not be measured.

Sample fabrication of planar hybrid samples: In a first step, one half of the as-prepared GaN layered samples (the sample discussed in the article and its reference samples discussed in Supplementary Note 1) were masked via spin-coating with a photoresist (AZ5214). The mask is patterned using lithography with rectangular holes with edge lengths of 100 µm, 200 µm, 300 µm and 400 µm. The masked samples are then transferred to the oCVD chamber. This step took about a week as the samples had to be transferred to a different lab. During the transfer, the samples were stored in darkness under ambient conditions. For the oCVD pro-

cess, the unmasked half of the samples is protected with Kapton tape. This ensures that PEDOT will cover just the masked part of the samples and therefore is at defined positions with defined sizes. The oCVD process was performed as described in the Materials Section of the main article. The samples were kept stable at $T_{Substrate}$ = 100°C during the growth process. The deposition time was $t_{deposition}$ = 630 s and the temperature of the oxidizing agent $FeCl_3$ was $T_{Oxidant}$ = 180°C. Immediately after the deposition process, the samples were rinsed using methanol and stored under nitrogen atmosphere. One week later the Kapton tape is removed, the previously unmasked half of the samples is masked with the same pattern like before (rectangular holes with edge lengths of 100 µm, 200 µm, 300 µm and 400 µm), and the contacts, 100 nm Au on the PEDOT and 30 nm Ti and 100 nm Au on the n-GaN half of the samples are deposited using thermal evaporation. Finally, the masks were removed using acetone and an ultrasonic bath. The samples were stored under ambient conditions in darkness.

Sample fabrication of planar LED sample: The LED substrate was grown via MOVPE on a 2'' sapphire wafer using the procedure discussed above. To set the back contact on the n-GaN, first, one part of the sample was etched down using reactive-ion etching with inductively coupled plasma (RIE-ICP) whilst the other part of the sample was protected using a chromium mask. The mask was etched of the sample and after cleaning the surface, n-contact pads with a thickness of 30 nm Ti and 300 nm Au using a shadow mask and thermal evaporation were deposited. To protect the etched area during the polymer deposition process, the sample was partially covered using Kapton tape. After the oCVD process as described above ($T_{substrate}$ = 180°C, $t_{deposition}$ = 660 s, $T_{monomer}$ = 160°C), the sample was rinsed using methanol. Contacts were deposited using a shadow mask and thermal evaporation. The quadratic contacts consist of a 10 nm palladium layer on which 300 nm Au is deposited with an edge length of 250 µm. Finally, the PEDOT layer is patterned in squares with three different quadratic sizes (edge length of 1 mm, 1.25 mm and 1.5 mm) using laser ablation. For that, the beam of a regenerative amplifier (520 nm wavelength, 336 fs pulse width, 200 kHz repetition rate) is scanned across the sample surface by a galvanometer scanner and focused by a telecentric objective, using pulse energy densities of 0.2 J cm^{-2} and several scan repetitions.

Sample fabrication of microrod structures: Hybrid GaN/PEDOT microrod structures needed no further processing steps. The as-prepared GaN microrods were placed in the oCVD vacuum chamber and the oCVD growth process ($T_{substrate}$ = 125°C, $t_{deposition}$ = 1200 s) followed the protocol discussed in the main article with a post-deposition 2-propanol rinsing step.

Sample fabrication of microrod LED structures: The as-prepared GaN microrod LED structures were coated using oCVD at standard conditions ($T_{substrate}$ = 140°C, $t_{deposition}$ = 1800 s) followed by five minutes post-deposition MeOH rinsing step. To reduce the risks of shunts due to broken microrods during the growth and sample preparation process, the microrod array was patterned using laser ablation to squared array fields with an edge length of ~75 µm. As for the hybrid planar LED structure, the beam of a regenerative amplifier (520 nm wavelength, 336 fs pulse width, 200 kHz repetition rate) is scanned across the sample surface by a galvanometer scanner and focused by a telecentric objective, using pulse energy densities of 0.2 J cm^{-2} and several scan repetitions.

Batch reproducibility: To ensure batch reproducibility and comparability, two approaches were taken. On the one hand, the oCVD process for the planar sample (discussed in Fig. 2 and 3) contained two samples of each doping concentration (discussed in Supplementary Note 1) that were placed inside the vacuum chamber. On the other hand, each sample was masked in such a way that multiple diodes were present on every single sample. Therefore, multiple diodes on each sample were measured at room temperature to ensure the diode formation over the whole sample. For the temperature-dependent I-V measurements, the sample presented as well as its equivalent sample were characterized and showed good agreement (Supplementary Fig. 2). Similarly, multiple hybrid LEDs from the same oCVD batch were obtained by structuring the individual planar and microrod LED samples into squares using laser ablation. Therefore, several devices with nearly identical PEDOT thin films could be compared regarding their EL and IV characteristics.

Supplementary References

1. Schmitsdorf, R. F., Kampen, T. U. & Mönch, W. Explanation of the linear correlation between barrier heights and ideality factors of real metal-semiconductor contacts by laterally nonuniform Schottky barriers. *J. Vac. Sci. Technol. B Microelectron. Nanom. Struct. Process. Meas. Phenom.* **15**, 1221–1226 (1997).
2. Werner, J. H. & Güttler, H. H. Barrier inhomogeneities at Schottky contacts. *J. Appl. Phys.* **69**, 1522 (1991).
3. Tung, R. T. Electron transport at metal-semiconductor interfaces: General theory. *Phys. Rev. B* **45**, 13509–13523 (1992).
4. Hartmann, J. *et al.* Growth mechanisms of GaN microrods for 3D core-shell LEDs: The influence of silane flow. *Phys. Status Solidi Appl. Mater. Sci.* **212**, 2830–2836 (2015).
